# Mercaptopurine Treatment in an Adult Man with Orbital and Intracranial Rosai-Dorfman Disease

**DOI:** 10.1155/2016/1030478

**Published:** 2016-10-20

**Authors:** Valentina Arnao, Marianna Riolo, Giovanni Savettieri, Paolo Aridon

**Affiliations:** Dipartimento di Biomedicina Sperimentale e Neuroscienze Cliniche, Università degli Studi di Palermo, Palermo, Italy

## Abstract

*Background*. Rosai-Dorfmann disease (RDD) is a rare, idiopathic non-Langerhans cell histiocytosis, affecting children and young adults, that commonly presents as painless, massive cervical lymphadenopathy with fever, weight loss, and polyclonal hypergammaglobulinemia. Cervical lymphadenopathy and extranodal involvement are the main presentations. On the contrary, ophthalmic involvement and localisation in the central nervous system are rare.* Case Report*. An old man was admitted to our hospital for first seizure. Brain imaging studies revealed on the left an extra-axial thickening of the dura mater with enhancement and perilesional oedema, infiltrating the sphenoorbital fissure and an isointense mass with enhancement in the orbital region with dislocation of the optic nerve. Pathological and immunohistochemistry examination of the bioptical specimen was consistent with a diagnosis of RDD. Treatment with levetiracetam and steroids was started obtaining only remission of seizures. Because of the patient refusal of the surgical debulking, therapy with mercaptopurine was started, stopping disease progression.* Conclusion*. So far, very few cases of extranodal RDD with multiple CNS lesions involving the orbital region have been described. Our case is significant because it is the first case in which the efficacy of mercaptopurine treatment has been documented in an adult patient with isolated ocular and intracranial RDD.

## 1. Introduction

Rosai-Dorfmann disease (RDD) is a rare idiopathic non-Langerhans cell histiocytosis, of unknown aetiology commonly presenting as painless, massive cervical lymphadenopathy with fever, weight loss, and polyclonal hypergammaglobulinemia. The disease mainly affects children and young adults [1, 2]. Over 90% of patients present with cervical lymphadenopathy. Extranodal involvement occurs in 40% of cases with anatomic distribution that include paranasal sinuses, respiratory tract, skin, nose, and bone. Ophthalmic involvement is seen in 10% of cases [3]. These include eyelid and orbital mass and rarely uveitis. Rosai-Dorfmann disease could mimic lymphoma, histiocytic and lacrimal gland tumours [3–7]. Localisation in the central nervous system (CNS) is rare (4% of cases) [8, 9]. Various treatments have been proposed, including steroid therapy, chemotherapeutic regimens, radiotherapy, surgery, and combinations of the above but optimal treatment has yet to be established.

## 2. Case Report

**Figure 1:**
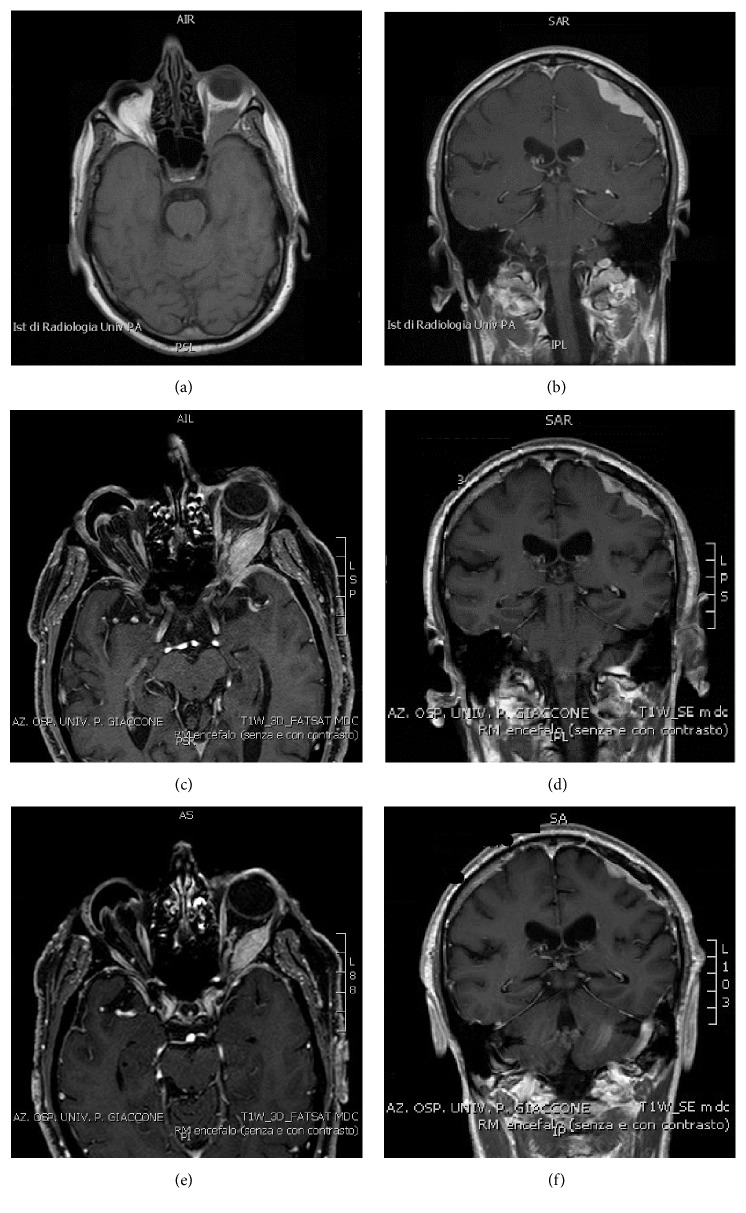
Axial (a, c, e) and coronal (b, d, f) enhanced T1 MR images reveal enhancing masses along the left frontoparietal-temporal region and the orbital region with dislocation of the optic nerve at onset (a, b) and in one-year (c, d) and two-year (e, f) follow-up, respectively.

A 70-year-old man, with history of hypertension, presented an abrupt onset of tremor and jerking of his right arm and, after two days, he was admitted to our hospital for a critical sudden episode with secondary generalization with loss of consciousness, followed by amnesia about the event. For few months, he has been conscious of reduced vision and foreign body sensation in his left eye. He also complained of dizziness, clumsiness, and paraesthesia in his right arm especially while using little objects and playing the piano. His family history was positive for tumours. When he was five years old, his right eye was enucleated because of an eye infection and, since then, he carries a prosthetic eye. He had a moderate low vision (20/160) in his left eye. Neurological examination showed distal weakness in his right arm. Brain CT scan showed slight hyperdensity in the left frontoparietal region, suggesting a tumour. Brain and orbit MRI revealed in left frontoparietal-temporal region extra-axial thickening of the dura mater with enhancement and perilesional oedema, infiltrating the sphenoorbital fissure, and an isointense mass with enhancement in the orbital region with dislocation of the optic nerve, which was thought to be consistent with a meningioma (Figures 1(a) and 1(b)). EEG shows diffuse slowing with excessive delta and theta activity. Blood examination was normal, except for hyperlipidemia and mild elevation of erythrocyte sedimentation rate. The patient underwent surgery to perform cerebral biopsy. Histopathological evaluation of the surgical piece revealed a fibrotic tissue with a histiocytic reaction and a large number of histiocytes containing normal-appearing lymphocytes within their cytoplasm (emperipolesis). Immunohistochemistry was positive for S-100 protein and CD68 leucocyte antigens and negative for CD1a. According to published criteria, these findings were consistent with a diagnosis of Rosai-Dorfman disease. The patient refused the surgical debulking to salvage his vision. Treatment with levetiracetam (500 mg twice a day) and steroid (Dexamethasone 4 mg intravenously once a day) was started with remission of critical symptoms. MRI features showed a decreased of edema but no modification of the lesions previously reported.

Three months later, a therapy with mercaptopurine was started with a daily dosage of 2,5 mg/kg, and steroid treatment was stopped. One year later, MRI showed slight reduction of the parietal-temporal lesion (Figures 1(c) and 1(d)), and levetiracetam therapy was stopped. At his one-year follow-up appointment, the patient reported no seizure activity without further loss of vision. The third brain MRI, performed approximately two years after diagnosis, showed a further reduction of the parietal-temporal lesion and of the left intra-orbital mass that enhanced uniformly after gadolinium administration (Figures 1(e) and 1(f)).

## 3. Discussion

Sinus histiocytosis with massive lymphadenopathy was originally described by Rosai and Dorfman in 1969 [1]. This is a rare non-Langerhans histiocytosis of unknown aetiology that usually presents with painless bilateral cervical lymphadenopathy, fever, anemia, leukocytosis, an elevated erythrocyte sedimentation rate, and polyclonal hypergammaglobulinemia. The ophthalmic involvement of Rosai-Dorfman disease include eyelid, orbital, and lacrimal gland manifestations and uveitis. Orbital involvement is the most common of ophthalmic manifestations [3–6]. Recently, also a case of compressive neuropathy of the optic nerve (with consecutive gradual vision loss) due to a RDD mass in ethmoid and sphenoid sinus extending into suprasellar region and causing bone erosion has been described [7].

Involvement of the central nervous system is rare [8, 9] and it occurs generally in middle-aged men (mean 39,4 years) causing headaches, seizure, visual symptoms, and focal deficits [9–12]. Involvement of the spinal cord has also been described [13, 14]. The disease is frequently reported in leptomeninges and it typically presents as a meningioma-like, extra-parenchymal, dural-based mass, similar, on MRI, to meningioma histiocytosis X lymphoproliferative disorders, plasma cell granulomas, and infectious disease [15, 16]. Nevertheless, there are also cases of isolated CNS presentation without dural involvement [17].

Histological and immunophenotypic assays are useful in establishing the diagnosis. In fact, RDD is characterized by abundant sheets of large and medium sized vacuolated histiocytes in a fibrous stroma, interspersed with foci of chronic inflammatory cells. In addition, the presence of emperipolesis is a hallmark of the disease, even if this may be less marked in intracranial disease [18]. In RDD, the histiocytes typically stain positive for S100 and CD68 and negative for CD1a [18–21]. These features with the absence of eosinophils and Birbeck's granules on electron microscopy make the differential diagnosis with CNS Langerhans Cell Histiocytosis (LCH) possible [18, 22, 23]. Another interesting differential diagnosis is Erdheim-Cester Disease (ECD), which is a rare non-Langerhans histiocytic disorder most commonly characterized by multifocal osteosclerotic lesions of the long bones. Neurologic involvement (mainly periorbital) is seen in 40 to 50 percent of cases. Biopsies of involved tissues are characterized by tissue infiltration by foamy (xanthomatous) histiocytes with interspersed inflammatory cells, multinucleate giant cells (Touton cells), and admixed or surrounding fibrosis. ECD cells express the histiocyte marker CD68, CD163, and Factor XIIIa but unlike Langerhans cell histiocytosis do not express CD1a or S100. Birbeck granules are absent (http://www.uptodate.com/).

In our case, the findings of emperipolesis, positivity for CD68 and S100 and negativity for CD1a were coherent with the diagnosis of RDD.

Most patients experience a course of spontaneous exacerbations and remissions [15]. However, if in 50% of systemic cases the disease will resolve spontaneously, a small percentage of patients (17%) will have asymptomatic persistent adenopathy and some will have residual symptoms for 5 to 10 years after onset [15]. Even if the course of intracranial lesions of RDD is generally considered to be benign, no spontaneous regression has been reported [1, 14]. Rarely intracranial RDD has an aggressive course [14].

Although a variety of treatments have been proposed [11, 13, 24], for the rare primary orbital and intracranial RDD only surgical resection, radical if possible, is considered the optimal treatment [25–27]. When this approach is not possible, according to the literature, roentgen therapy, chemotherapy, steroid therapy [11, 28], rituximab [29], interferon-alfa-2a [30], and immunosuppressive agents, such as azathioprine, methotrexate, and mercaptopurine [31–33], can be used.

Alqanatish et al. described a case of a 7-year-old child with concomitant LES and RDD, who was treated with rutiximab (500 mg/m^2^/dose), with complete remission of the massive lymphadenopathy after 7 weeks of treatment [29]. Also high dose of interferon-alfa-2a, in particular, the pegylated form, has shown dramatic efficacy in cases of systemic RDD [30]. Nevertheless, it should be avoided because it could favour the occurrence of seizures [31]. Other options include immunosuppressive agents, such as azathioprine. Le Guenno et al. described a 57-year-old man with a history of diseases that involve the monocyte/macrophage system (Q fever and Crohn disease) who developed also RDD and was successfully treated with azathioprine [32]. Moreover, two children showed a sustained response to a combination of methotrexate and mercaptopurine [33, 34]. Another two paediatric cases with nonintracranial involvement showed a good response to chemotherapy with vinblastine, prednisone, 6-mercaptopurine and methotrexate [35], and 2-chlorodeoxyadenosine (2-CdA, cladribine) [36].

Like a minority of patients with concurrent orbital and neurological manifestations as the sole extranodal site of involvement without synchronous nodal disease, surgery could have represented the best therapy for our patient; unfortunately, he refused this option. Therefore, considering the failure of conventional approach with steroid therapy, a treatment with mercaptopurine was started. This is a purine antagonist, which inhibits DNA and RNA synthesis acting as a false metabolite. The treatment caused a stabilization of the lesions and after 24 months, no adverse event has been reported including anemia, granulocytopenia, haemorrhage, lymphocytopenia, and leukopenia. Although spontaneous remissions are not uncommon and surgical resection appears to be the most efficacious approach, therapy with mercaptopurine in our patient led to stopping of the disease progression. To our knowledge, considering that the other reported cases are paediatric [33–36], this is the first reported case of an adult patient with isolated ocular and intracranial RDD in which the efficacy of mercaptopurine treatment has been documented. In this way, our case supports the use of purine antagonists and purine antimetabolite for an efficacious treatment of adult forms of RDD.
